# Systemic hydrocortisone to prevent bronchopulmonary dysplasia in preterm infants (the SToP-BPD study); a multicenter randomized placebo controlled trial

**DOI:** 10.1186/1471-2431-11-102

**Published:** 2011-11-09

**Authors:** Wes Onland, Martin Offringa, Filip Cools, Anne P De Jaegere, Karin Rademaker, Henry Blom, Eric Cavatorta, Anne Debeer, Peter H Dijk, Arno F van Heijst, Boris W Kramer, Andre A Kroon, Thilo Mohns, Henrica L van Straaten, Arjan B te Pas, Claire Theyskens, Mirjam M van Weissenbruch, Anton H van Kaam

**Affiliations:** 1 Department of Neonatology, Emma Children's Hospital, Academic Medical Center, Amsterdam, the Netherlands; 2 Department of Paediatric Clinical Epidemiology, Emma Children's Hospital, Academic Medical Center, University of Amsterdam, the Netherlands; 3 Department of Neonatology, Universitair Ziekenhuis Brussel, Brussel, Belgium; 4 Department of Neonatology, Wilhelmina Children's Hospital, University Medical Center Utrecht, Utrecht, the Netherlands; 5 Department of Neonatology, Universitair Ziekenhuis Antwerpen, Antwerpen, Belgium; 6 Department of Neonatology, Centre Hospitalier Universitaire de Charleroi, Charleroi, Belgium; 7 Department of Neonatology, Universitair Ziekenhuis Leuven, Leuven, Belgium; 8 Department of Neonatology, University Medical Center Groningen, Groningen, The Netherlands; 9 Department of Neonatology, Radboud University Nijmegen Medical Center, Nijmegen, The Netherlands; 10 Department of Neonatology, Maastricht University Medical Center, Maastricht, The Netherlands; 11 Department of Neonatology, ErasmusMC-Sophia, Rotterdam, The Netherlands; 12 Department of Neonatology, Maxima Medical Center Veldhoven, the Netherlands; 13 Department of Neonatology, Isala Clinics, Zwolle, The Netherlands; 14 Department of Neonatology, Leiden University Medical Center, Leiden, The Netherlands; 15 Department of Neonatology, Ziekenhuis Oost-Limburg, Genk, Belgium; 16 Department of Neonatology, VU University Medical Center, Amsterdam, The Netherlands

## Abstract

**Background:**

Randomized controlled trials have shown that treatment of chronically ventilated preterm infants after the first week of life with dexamethasone reduces the incidence of the combined outcome death or bronchopulmonary dysplasia (BPD). However, there are concerns that dexamethasone may increase the risk of adverse neurodevelopmental outcome. Hydrocortisone has been suggested as an alternative therapy. So far no randomized controlled trial has investigated its efficacy when administered after the first week of life to ventilated preterm infants.

**Methods/Design:**

The SToP-BPD trial is a randomized double blind placebo controlled multicenter study including 400 very low birth weight infants (gestational age < 30 weeks and/or birth weight < 1250 grams), who are ventilator dependent at a postnatal age of 7 - 14 days. Hydrocortisone (cumulative dose 72.5 mg/kg) or placebo is administered during a 22 day tapering schedule. Primary outcome measure is the combined outcome mortality or BPD at 36 weeks postmenstrual age. Secondary outcomes are short term effects on the pulmonary condition, adverse effects during hospitalization, and long-term neurodevelopmental sequelae assessed at 2 years corrected gestational age. Analysis will be on an intention to treat basis.

**Discussion:**

This trial will determine the efficacy and safety of postnatal hydrocortisone administration at a moderately early postnatal onset compared to placebo for the reduction of the combined outcome mortality and BPD at 36 weeks postmenstrual age in ventilator dependent preterm infants.

**Trial registration number:**

Netherlands Trial Register (NTR): NTR2768

## Background

Bronchopulmonary dysplasia (BPD) is the most common complication of premature birth, with a reported incidence of 8% to 35% [1,2]. BPD is characterized by chronic respiratory distress, the need for prolonged respiratory support, an increased risk of recurrent pulmonary infections leading to hospital readmissions during the first years of life [3], and life-long alterations in lung function [4-6]. BPD is also considered an important risk factor for adverse neurodevelopmental outcome after premature birth [7-11]. These long-term consequences of BPD place a heavy burden on societal and health care costs [12-15].

Pulmonary inflammation has been identified as an important risk factor in the development of BPD, providing the rationale for treating patients at risk for BPD with glucocorticoids, a group of drugs well known for its strong anti-inflammatory effect [16-18]. Randomized controlled trials (RCTs) comparing dexamethasone to placebo in ventilated preterm infants at risk for BPD have shown that dexamethasone reduces the incidence of the combined outcome death or BPD [19,20]. Dexamethasone seems to be most effective when initiated between 7 to 14 days postnatal age, the so-called moderately early treatment onset [21,22]. Unfortunately, more recent follow-up studies seem to indicate that this beneficial effect on the lung may be outweighed by an increased risk of adverse neurodevelopmental outcome. Despite the fact that this adverse effect on the newborn brain has so far only been reported in studies investigating the early use of dexamethasone (i.e. initiated < 7 days of postnatal age), there is now a general concern on its use in preterm infants [23-25]. Based on this concern, the American Academy of Pediatrics, Canadian Paediatric Society, and the European Association of Perinatal Medicine have stated that the use of dexamethasone should be restricted to exceptional cases and clinical trials should be performed to investigate the use of alternative anti-inflammatory glucocorticoids, such as hydrocortisone [26,27].

In response to this statement the use of dexamethasone has dropped dramatically to approximately 10% of the preterm infants at risk for BPD [28-30]. In addition, its dose has been significantly reduced and administration is often postponed until the 3^rd ^or 4^th ^week of life [23].

Some clinicians have started to use hydrocortisone, as an alternative for dexamethasone. Animal studies have suggested that, in contrast to dexamethasone, hydrocortisone has no detrimental effect on the brain [31]. To date, eight RCTs including 880 preterm infants investigated a low hydrocortisone dose started within 72 hours after birth (early treatment onset). Meta-analysis of these trials failed to show a clear reduction in the incidence of death or BPD [32]. Only one of these trials reported long-term follow-up, showing no differences in adverse neurodevelopmental sequelae [33].

To our knowledge, there are no placebo controlled randomized trials that investigated the use of hydrocortisone after the first week in life in ventilator dependent preterm infants [34] The neonatal intensive care unit (NICU) at the University Medical Center Utrecht has historically used hydrocortisone after the first week of life for ventilated preterm infants at high risk for developing BPD. Retrospective cohort studies conducted in this center seem to indicate that hydrocortisone is effective in reducing BPD, without causing serious long-term adverse effects [35-37]. However, these findings need to be confirmed or refuted by a large randomized placebo controlled trial.

### Aim of the study

To investigate if systemic hydrocortisone, started between 7 - 14 days after birth, is safe and effective in reducing the incidence of the combined outcome death or BPD at 36 weeks PMA in chronically ventilated preterm infants, as compared to a placebo.

### Study design

Multicenter randomized double-blind placebo-controlled trial conducted in 15 NICUs in the Netherlands (n = 10) and Belgium (n = 5). The inclusion period will be 3 years, with a 2 year follow-up for each child.

### Study population

#### Inclusion criteria

Preterm infants with:

- gestational age < 30 wks and/or birth weight < 1250 g

- ventilator dependency at 7-14 days PNA

- respiratory index (RI), defined as the product of mean airway pressure (MAwP) and the fraction of inspired oxygen (FiO_2_), of ≥ 3.5 for more than 12 h/day for at least 48 hours, ensuring adequate oxygen saturation (85-95%) and PaCO_2 _values (5.0-7.5 kPa).

#### Exclusion criteria

- chromosomal defects (e.g. trisomy 13, 18, 21)

- major congenital malformations that:

○ compromise lung function (e.g. surfactant protein deficiencies, congenital diaphragmatic hernia)

○ result in chronic ventilation (e.g. Pierre Robin sequence)

○ increase the risk of death or adverse neurodevelopmental outcome (e.g. congenital cerebral malformations)

- use of dexamethasone or hydrocortisone prior to inclusion for the sole purpose of improving lung function and respiratory status

#### Sample size calculation

Estimating the a priori risk of death or BPD in preterm infants less than 30 weeks gestation and ventilated in the second week of life at 60 - 70% and defining an absolute risk reduction of 15% or more (NNT = 7) as clinically relevant, 175 patients need to be included in each treatment arm (type I error of 5%, power of 80%). Anticipating a 10% drop out of randomized patients, the number of inclusions needs to be increased to 200 patients in each arm (total 400 patients).

### Treatment of subjects

#### Trial medication and dosing

Trial medication will be prepared and distributed according to Good Clinical Practice (GCP) and Good Manufacturing Practice (GMP) guidelines. Using a double-blinded treatment design, infants will be allocated to either hydrocortisone or placebo treatment.

The optimal (cumulative) dose of hydrocortisone is currently unknown. Pharmacokinetic studies have not been done, and are tedious in this field of developmental endocrinology. However, the NICU of the University Medical Center in Utrecht (the Netherlands) has used a fixed hydrocortisone treatment regimen for several decades, which has recently been adopted by 4 other Dutch NICUs. Retrospective cohort studies have suggested that this treatment regimen is equally effective in reducing death or BPD compared with dexamethasone treated infants without causing an increased risk of adverse neurological outcome [36,38]. Based on these findings and current clinical practice in the Netherlands, we decided to adopt the dosing regimen from Utrecht for this study. Infants in the hydrocortisone group will be treated with hydrocortisone 5 mg/kg/day Q.I.D for 7 days, followed by 3.75 mg/kg/day T.I.D. for 5 days, subsequently lowering the frequency by one dose every 5 day. This leads to a total duration of therapy of 22 days and a cumulative dose of 72.5 mg/kg hydrocortisone. The infants in the control group receive a placebo treatment for the entire 22-day period in the same frequency as the hydrocortisone group. Both hydrocortisone and placebo schedules will be calculated according to weight on the day of randomization and not adjusted to the actual weight during the tapering schedule.

#### Treatment failure and use of open label hydrocortisone

In general, the use of open label hydrocortisone during the 22 day treatment course is strongly discouraged. Open label hydrocortisone use **may be considered **in the following conditions:

1. The pulmonary condition is progressively deteriorating and the RI > 10 for more than 6 consecutive hours

2. The pulmonary condition of the patient is stable (RI ≤ 10) but not improving over time. If this is the case, open label hydrocortisone **may be considered **if both of the following conditions are met:

a. Extubation was attempted (extubation trial) within 24 hours before considering open label treatment and this attempt failed

b. The patient has been on study medication for **at least **10 days

If, based on the aforementioned criteria, the physician decides to start open label hydrocortisone, the study medication is stopped and the patient will be recorded as "treatment failure". The open label hydrocortisone dosage schedule is similar to that used in the study. In case of treatment failure the following data will be collected: timing of treatment failure, ventilator support and settings, type of open label medication, starting date, cumulative dose and duration of open label therapy. These patients will be followed as all other patients.

#### Late rescue hydrocortisone therapy

If a patient is still on mechanical ventilation after completion of the study medication (i.e. day 22) and a subsequent extubation attempt failed, the physician may consider treatment with late rescue hydrocortisone. The late rescue hydrocortisone dosing schedule is similar to that used in the study. Data on the starting date, cumulative dose and duration of rescue therapy are collected. The patients will be followed as all other patients.

#### Hydrocortisone for hypotension

In case of persistent hypotension, not (sufficiently) responding to first line treatment with intravascular volume expansion and inotropes (i.e. dopamine and/or dobutamine) the use of hydrocortisone is allowed in a dose of 3 mg/kg/day for 5 days [39]. The use of hydrocortisone for this indication will not be considered as treatment failure. Data on timing, dose and duration will be collected.

#### Inhalation corticosteroids

The use of inhaled corticosteroids in the first weeks of life is strongly discouraged. However, its use is not an exclusion criterion, because there is currently insufficient evidence that inhaled corticosteroids will reduce the risk of death or BPD in preterm infants. Data on timing, dose and duration will be collected.

#### Use of co-intervention

All randomized patients will be subjected to co-interventions, such as respiratory support and co-medication, according to the guidelines and protocols of the individual NICUs.

## Methods/Design

### Randomization

Eligible patients can be randomized between day 7 and 14 PNA. The first dose of study medication should be administered within 24 hours after randomization. Randomization will be centrally controlled and web-based using a computer program designed for this study. This trial will be protected from selection bias by using concealed, stratified and blocked randomization. Randomization will be stratified per center and according to gestational age stratum (stratum A: < 27 weeks; stratum B: **≥ **27 weeks) and the allocation ratio will be 1:1 using variable block sizes. Multiple birth infants will be randomized independently, unless the parents or caregivers explicitly request that the siblings are allocated to the same treatment arm. In case of the latter the randomization software will be able to perform twin randomization. The infants' parents and all members of the medical team, including investigators, remain blinded to group assignment throughout the study.

### Withdrawal of individual subjects

Parents or caregivers may decide to withdraw their infant from the study at any time and without any consequences. Subjects withdrawn from the study will be treated according to the standard of care, including neurodevelopmental outcome assessment at the outpatient clinic. To ensure adequate inclusion, withdrawn patients will be replaced by additional inclusions.

### Outcomes

#### Primary outcome

The primary outcome will be the dichotomous combined outcome death or BPD at 36 weeks PMA. BPD at 36 weeks PMA will be assessed according to the NICHD Consensus Statement using the classification of severity as proposed by Jobe et.al. [18] and if indicated the oxygen reduction test as described by Walsh et.al. [18,40,41].

#### Secondary outcomes before hospital discharge

• treatment failure

• mortality at 28 days PNA, 36 weeks PMA and at hospital discharge

• BPD at 28 days

• failure to extubate 3, 7, 14 and 21 days after initiating therapy

• duration of mechanical ventilation

• use of late rescue hydrocortisone treatment

• total time on supplemental oxygen

• length of hospital stay

• hypertension, as previously defined

• hyperglycemia requiring the use of insulin therapy

• nosocomial infection, including clinical or culture proven sepsis, meningitis and pneumonia

• patent ductus arteriosus for which medical intervention or surgical ligation is needed

• necrotising enterocolitis (NEC) with Bell stage II or more

• gastrointestinal bleeding

• spontaneous intestinal perforation

• intraventricular haemorrhage (IVH) and/or periventricular leucomalacia (PVL), including grading on cerebral ultrasonography according to protocol as defined by Ment et.al. [42]

• retinopathy of prematurity, including grading according to international classification [43]

• weight, head circumference and length at 36 weeks PMA

#### Secondary outcomes after hospital discharge

• mortality

• number of readmissions since first discharge home

• weight, length and head circumference at 24 months corrected age (c.a.)

• Bayley Scales of Infant Development III, Mental Developmental Index and Psychomotor Developmental Index at 24 months c.a.

• cerebral palsy and severity of cerebral palsy using gross motor function classification system at 24 months c.a.

• hearing loss requiring hearing aids at 24 months c.a.

• blindness at 24 months c.a.

• behavioral problems (child behavior checklist) at 24 months c.a.

### Data collection and statistical analysis

At the time of inclusion and randomization, baseline data will be collected from all patients, including maternal and patient characteristics, respiratory status and support, and the presence of serious morbidity as listed in the secondary outcomes. During the 22 d treatment course, respiratory support, blood gas analyses, serum glucose levels, blood pressure and anthropometric data will be collected on a daily bases. Outcome variables and co-interventions will be recorded at 36 weeks PMA, hospital discharge and at follow-up (2 years corrected age).

Data on eligible patients not included in the study will also be recorded, including patient characteristics, respiratory status and support, and the reason why the patient was not included in the trial.

Data will be presented as mean ± standard deviations or medians and (interquartile) ranges depending on their distribution. Categorical data will be analyzed using the Chi-square test. Continuous data will be analyzed using the Student's *t *test or Mann-Whitney test as appropriate. Both intention-to-treat and per-protocol analysis will be employed since use of open label glucocorticoids may modulate possible treatment effects [44]. The effect of hydrocortisone on the primary outcome BPD free survival will be assessed by multi-variable logistic regression analysis including possible confounders. Statistical significance is set at p < 0.05.

### Safety aspects and study monitoring

During the study all patients will be closely monitored for adverse events. First, complications of preterm birth and intensive care treatment, as listed in the outcome section of this study protocol, will be reported once a year to the Data Monitoring Committee (DMC) and the Ethics Committee that approved the study protocol. Second, all adverse events, not listed as outcomes in this study will be reported within the appropriate time frame to the Data Monitoring Committee (DMC) and the Ethics Committee that approved the study protocol. Finally, to enhance safety of this study the principle investigator and the study coordinator will be automatically informed by email in case a patient

1) is treated with indomethacin or ibuprofen while receiving study medication;

2) is diagnosed as having an intestinal perforation;

3) is diagnosed as having hypertension; and

4) receives open label hydrocortisone.

In addition to the above, the DMC will monitor efficacy and safety outcomes and will provide the trial's Steering Committee with recommendations regarding continuing or stopping the trial (for all patients or subgroups of patients) when approximately 25% (safety only), 50% (safety and efficacy) and 75% (safety and efficacy) of the anticipated outcome data are available. The DMC will be blinded to the treatment allocation, but may request the data manager to reveal the allocation labels.

The study will be monitored by means of personal visits to the investigators' facilities and through other communications (e.g., telephone calls, written correspondence). The main goal of monitoring is to evaluate the progress of the study, ensure the rights and wellbeing of the subjects are protected, check that the reported clinical study data are accurate, complete and verifiable from source documents, and the conduct of the study is in compliance with the approved protocol and amendments, GCP and applicable national regulatory requirements. A monitoring visit will include a review of the essential clinical study documents (regulatory documents, CRFs, source documents, drug disposition records, subject informed consent forms, etc.) as well as discussion on the conduct of the study with the investigator and staff.

In case of hypertension defined as a systolic blood pressure > 80 mmHg for infants with a gestational age of 24-26 wks, > 90 mmHg for infants 26-28 wks, and > 100 mmHg for infants ≥ 28 wks, the hydrocortisone dose is lowered according to a predefined tampering schedule. Data on the time, reason and dose adjustment will be collected.

### Ethical considerations

The study will be conducted according to the principles of the Declaration of Helsinki [45] and in accordance with the Medical Research Involving Human Subjects Act (WMO). Patients can only be included in the study after obtaining written informed consent from both parents. Both parents will receive verbal and written information on all aspects of the study, including possible risks and benefits. The right of a parent to refuse participation without a given reason will be respected. The parents will remain free to withdraw their child at any time from the study without consequences for further treatment. The study protocol has been reviewed and approved by the Ethics Committee of the Academic Medical Center in Amsterdam (the Netherlands) and the local Ethics Committee of each participating hospital.

### Handling and storage of data and documents

Data management will be implemented according to GCP guidelines. Patient data will be entered via an electronic case record form (CRF) in a central GCP proof web-based database to facilitate on-site data-entry. Security is guaranteed with login names, login codes and encrypted data transfer. The data of all subjects will be coded and this coding will not be retraceable to the individual patient. The key to this coding is safeguarded by the investigator. Access will be granted to the principal investigator, local investigator, the study monitor, quality assurance auditor, the Ethics Committee and the inspector of the Ministry of Health. Data will be stored for 15 years.

## Abbreviations

BPD: BronchoPulmonary Dysplasia; CRF: Case Record Form; DMC: Data Monitoring Committee; FiO_2: _Fraction of Inspired Oxygen; GA: Gestational Age; GCP: Good Clinical Practice; GMP: Good Manufacturing Practice; IVH: IntraVentricular Haemorrhage; MAwP: Mean Airway Pressure; NEC: Necrotising EnteroColitis; NICU: Neonatal Intensive Care Unit; NICHD: National Institutes for Child Health and Human Development; NNT: Number Needed to Treat; PMA: Postmenstrual Age; PNA: Postnatal Age; PVL: Periventricular Leucomalacia; RCT: Randomised Controlled Trial; RI: Respiratory Index.

## Competing interests

The authors declare that they have no competing interests.

## Authors' contributions

WO, ADJ, MO, AvK were involved in drafting the conception and design of the study. All other authors were involved in the final consensus process of the protocol and contributed significantly to the final version. WO, ADJ, MO, AvK drafted the manuscript and all other authors read, edited and approved the final manuscript.

## Pre-publication history

The pre-publication history for this paper can be accessed here:

http://www.biomedcentral.com/1471-2431/11/102/prepub
